# Editorial: Inflammatory pain: mechanisms, assessment, and intervention

**DOI:** 10.3389/fnmol.2023.1286215

**Published:** 2023-09-28

**Authors:** Yong-Hui Zhang, Daniela Adamo, Howe Liu, Quanxing Wang, Wen Wu, Yi-Li Zheng, Xue-Qiang Wang

**Affiliations:** ^1^Department of Rehabilitation Medicine, Shanghai University of Medicine and Health Sciences Affiliated Zhoupu Hospital, Shanghai, China; ^2^Department of Sport Rehabilitation, Shanghai University of Sport, Shanghai, China; ^3^Department of Neuroscience, Reproductive and Odontostomatological Sciences, University of Naples Federico II, Naples, Italy; ^4^Department of Physical Therapy, Allen College, Waterloo, IA, United States; ^5^National Key Laboratory of Medical Immunology and Institute of Immunology, Second Military Medical University, Shanghai, China; ^6^Department of Rehabilitation, Zhujiang Hospital, Southern Medical University, Guangzhou, China

**Keywords:** inflammatory pain, neuropathic pain, inflammation, pain, function

Inflammatory pain refers to the increased sensitivity of perception and emotional response to noxious stimuli, that arises from an inflammatory reaction associated with tissue damage (Layne-Stuart and Carpenter, 2022). Under normal conditions, acute inflammation is a protective response by the body to tissue damage or infection, potentially leading to the perception of pain while damaged tissues are being cleared and repaired (Muley et al., 2016). Inflammatory pain can serve as a reminder of a recent injury to prevent re-injury and thereby achieve quick recovery. In general, inflammation management effectively relieves inflammatory pain due to the reduced stimulation of nerves following the resolution of inflammation. However, chronic inflammation can lead to adverse pain because inflammatory mediators act on pain-sensitive nerve endings by decreasing the thresholds of neuronal excitability and increasing the sensitivity of firing rates, thereby causing peripheral and central sensitization (Prescott and Ratté, 2017). Under these sensitized conditions, pain perception can be abnormal, such as allodynia (perceiving innocuous stimuli as painful) and hyperalgesia (amplifying the intensity and duration of pain caused by noxious stimuli) (Laverdure-Dupont et al., 2009). In clinics, chronic pain is normally a complex of inflammatory and neuropathic components. Inflammatory mediators can result in neuronal damage, which triggers an inflammatory reaction. These adverse and complex pain conditions lead to less physical activity, negative psychological state (anxiety and depression), low quality of life, and heavy economic burden. Understanding the mechanisms and interventions of inflammatory pain allows us to increase the efficacy of pain management and alleviate social burdens.

This research includes seven peer-reviewed published articles, namely, two bibliometric analyses regarding neuropathic pain, two articles discussing biomarkers of inflammatory pain, and three articles about the interventions for inflammatory pain (exercise, platelet-rich plasma injections, and shockwave therapy) (Figure 1). These articles provide novel evidence in this field and discuss the latest research trends, mechanisms, or interventions of inflammatory pain, and the future challenges and research direction for this topic.

**Figure 1:**
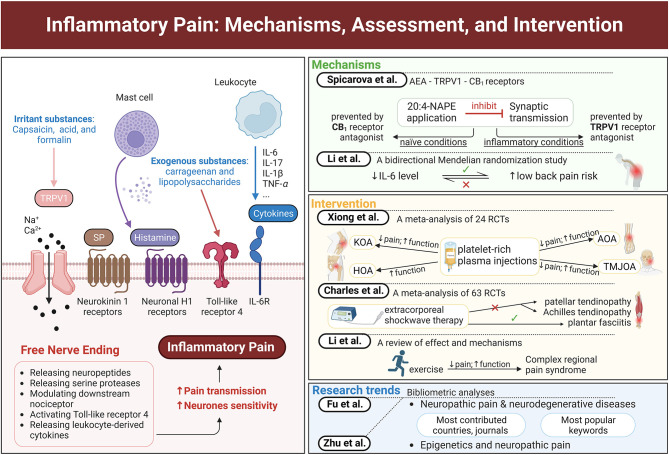
Graphical abstract of major concepts and themes explored in this Research Topic. The left panel shows peripheral sensitization of a free nerve ending by various inflammatory mediators and irritant substances. The right panel lists the main ideas of each study in this Research Topic. This figure was created with BioRender.com.

The general mechanism of inflammatory pain involves proinflammatory mediators, such as cytokines, proteases, neuropeptides, and growth factors, which are released around inflammation locations to sensitize peripheral pain-sensing neurons (Kidd and Urban, 2001; Lipnik-Stangelj, 2013) (Figure 1). Some irritant substances, such as capsaicin, acid, and formalin, can open transient receptor potential vanilloid type 1 (TRPV1) ion channels, to release neuropeptides, thereby increasing the sensitivity of neurons. N-arachidonoylphosphatidylethanolamine (20:4-NAPE) in enzyme preparations produces anandamide (AEA), which can activate TRPV1 and cannabinoid 1 (CB_1_) receptors (Wang et al., 2006). Spicarova et al. illustrate the inflammation-induced alterations in the function of AEA-TRPV1-CB_1_ receptors by investigating the influence of local AEA produced from 20:4-NAPE on the miniature excitatory postsynaptic currents in naïve and inflammatory conditions. They find that 20:4-NAPE has a significant inhibitory effect on spinal cord synaptic transmission, which is mediated by TRPV1 and CB1 receptors. This inhibition is prevented by CB1, not TRPV1, receptor antagonist under naïve conditions. Meanwhile, TRPV1, not CB1, receptor antagonist becomes responsible for this inhibition with peripheral inflammation. This switch may be one of the important mechanisms underlying pain development.

Inflammation plays an essential role in low back pain and spinal degeneration (Risbud and Shapiro, 2014). Hence, a bidirectional two-sample Mendelian randomization study was conducted by Li W. et al. to investigate the relationship between inflammatory biomarkers and low back pain. Their results indicate that low levels of inflammatory biomarkers IL-6, not IL-8, IL-10, or C-reactive protein, are associated with an increased risk of low back pain. On the contrary, no significant causal effect of low back pain on inflammatory biomarkers is found in the reverse direction. This evidence reminds researchers and clinicians that an increase in IL-6 levels may reduce the risk of low back pain.

Current interventions for inflammatory pain can be categorized as pharmacological and non-pharmacological. Except for analgesics, other treatments targeting different types of inflammatory pain are explored. Xiong et al. investigate the effect of platelet-rich plasma injections on different kinds of osteoarthritis, including knee osteoarthritis (KOA), hip osteoarthritis (HOA), ankle osteoarthritis (AOA), and temporomandibular joint osteoarthritis (TMJOA). They also conducted a systematic search and included 24 randomized controlled trials with 1,344 patients for the meta-analysis. Their results support that platelet-rich plasma injections are effective and safe for pain alleviation in patients with KOA, TMJOA, and AOA but not in those with HOA. These injections also improve the functional activity of all patients with osteoarthritis. Moreover, the efficacy of leukocyte-poor platelet-rich plasma injections for pain alleviation from osteoarthritis is better than that of leukocyte-rich platelet-rich plasma injections. The meta-analysis by Charles et al. reports another nonpharmacological treatment, shockwave therapy, for patellar tendinopathy, Achilles tendinopathy, and plantar fasciitis. Their meta-analysis including 63 randomized controlled trials provides high-quality evidence of the substantial efficacy of extracorporeal shockwave therapy (ESWT) in improving pain and function in patients with plantar fasciitis. In addition, low-moderate evidence confirms the limited efficacy of ESWT in alleviating pain and disability in patients with patellar tendinopathy and Achilles tendinopathy compared with placebo, eccentric exercise, or other treatments. These two meta-analyses provide additional information about the effect of platelet-rich plasma injections on different types of osteoarthritis and the effect of shockwave therapy on different kinds of tendinopathy.

Exercise, a popular topic in recent years, is another important nonpharmacological intervention for pain (Goebel et al., 2019; Harden et al., 2022). Li T.-S. et al. summarize the effects of exercise on complex regional pain syndrome, a chronic pain with inflammatory features. Their review indicates that exercise, such as graded motor imagery and progressive strength and aerobic training, is an effective intervention for pain alleviation and physical and mental function improvement among patients with complex regional pain syndrome. The authors also discuss the potential mechanisms of this effectiveness, including improving central and peripheral nervous system sensitization, regulating vasodilation and adrenaline levels, releasing endogenous opioids, and increasing anti-inflammatory cytokines levels. This mini-review provides meaningful information about this topic and points out future research directions.

Many research articles have discussed neuropathic pain and neurodegenerative diseases. Meanwhile, Fu et al. review the research trends and hot spots in this topic through a detailed search of the Web of Science Core Collection Database. Their bibliometric analysis includes 387 articles, of which 85.27% were published during 2007–2022. The top three discussed neurodegenerative diseases were multiple sclerosis, Parkinson's disease, and Alzheimer's disease. The mechanism research focused on microglia-regulated neuroinflammation, and the intervention research was concentrated on deep brain stimulation and gamma knife radiosurgery. The United States contributed the most to this topic, accounting for the highest number of studies, citations, and H-indexes. This evidence provides additional information about the research trends in neurodegenerative diseases and neuropathic pain.

For the topic of epigenetics and neuropathic pain, Zhu et al. search for related articles and reviews from the Science Citation Index-Expanded of the Web of Science Core Collection database and analyze the countries, journals, and keywords of the 867 included studies. China, the United States, and Japan were the top three countries that contributed the highest number of studies during 2007–2022. The top three journals publishing the most papers were Molecular Pain, Pain, and Journal of Neuroinflammation. The popular keywords in this research field were DNA methylation, circular RNA, acetylation, long non-coding RNA, and microglia. The authors report this information about epigenetics and neuropathic pain and provide further study directions for researchers in this field.

These seven articles published on this Research Topic include different types of research, providing novel evidence about inflammatory pain from different perspectives. Although some of them are slightly outside the scope of the topic, all the articles revolve around the global research trends, potential mechanisms, or interventions of inflammatory pain and help us to further understand inflammatory pain.

## Author contributions

Y-HZ: Visualization, Writing—original draft, Writing—review and editing. DA: Writing—review and editing. HL: Writing—review and editing. QW: Writing—review and editing. WW: Writing—review and editing. Y-LZ: Writing—review and editing. X-QW: Conceptualization, Supervision, Writing—review and editing.
